# Sudden cardiac death in a young athlete due to anomalous origin of both coronaries from a common ostium

**DOI:** 10.1007/s12024-025-00979-9

**Published:** 2025-04-02

**Authors:** Stefania Zerbo, Giuseppe Davide Albano, Ginevra Malta, Alberto Alongi, Emiliano Maresi, Antonina Argo

**Affiliations:** Section of Legal Medicine, Department of Health Promotion, Mother and Child Care, Internal Medicine and Medical Specialties, University of Palermo, Palermo, 90129 Italy

**Keywords:** Anomalous aortic origin of the coronary arteries, Autopsy, Forensic pathology, Sudden cardiac death, Athletes, Coronary arteries anomalies, Postmortem MRI

## Abstract

Sudden cardiac death (SCD) in young people is a tragic event and a worldwide public health issue. SCD is the most common cause of death in young athletes and is related to acquired/congenital cardiac disorders, such as cardiomyopathies, congenital coronary anomalies and ion channelopathies. We report the case of a 14-year-old male non professional athlete who was apparently healthy but suddenly died during a football game. His clinical history was negative for any significant medical illness. At autopsy, a congenital anomalous origin of both the right and left coronary arteries from a single aortic ostium above the aortic cusp was observed with a concomitant hypertophic cardiomyopathy (HCM) The toxicological analysis was negative for common drugs of abuse. The death was attributed to SCD due to hyperkinetic ventricular arrhythmia secondary to myocardial ischemia in a subject with an anomalous origin of both coronary arteries and underlying hypertrophic cardiomyopathy. This report highlights a rare congenital anomalous origin of the coronary arteries associated with a high risk of SCD, particularly in young athletes.

## Case report

A 14-year-old Caucasian male non professional athlete suddenly collapsed during a basketball game. He received cardiopulmonary resuscitation and defibrillation before being transferred to the emergency department, but he died in an ambulance. Legal authorities ordered an autopsy to investigate the cause of death.

According to his parents, he had been living a normal life. There was no history of any relevant medical illness or of chest pain, dyspnea, arrhythmias or other causes of cardiac disease. Furthermore, no other sudden death of a young family member was reported by his family. A complete autopsy was performed. The height of the decedent was 165 cm, and the weight was 56 kg. Upon external examination, discrete cervicobrachiocephalic congestion was observed. No traumatic lesions were detected.

Internal examination revealed cardiac hypertrophy, marked polyvisceral congestion and pulmonary edema. The heart weighed 320 g. The longitudinal and transverse diameters of the heart were 8.5 and 9 cm, respectively.

**Fig. 1:**
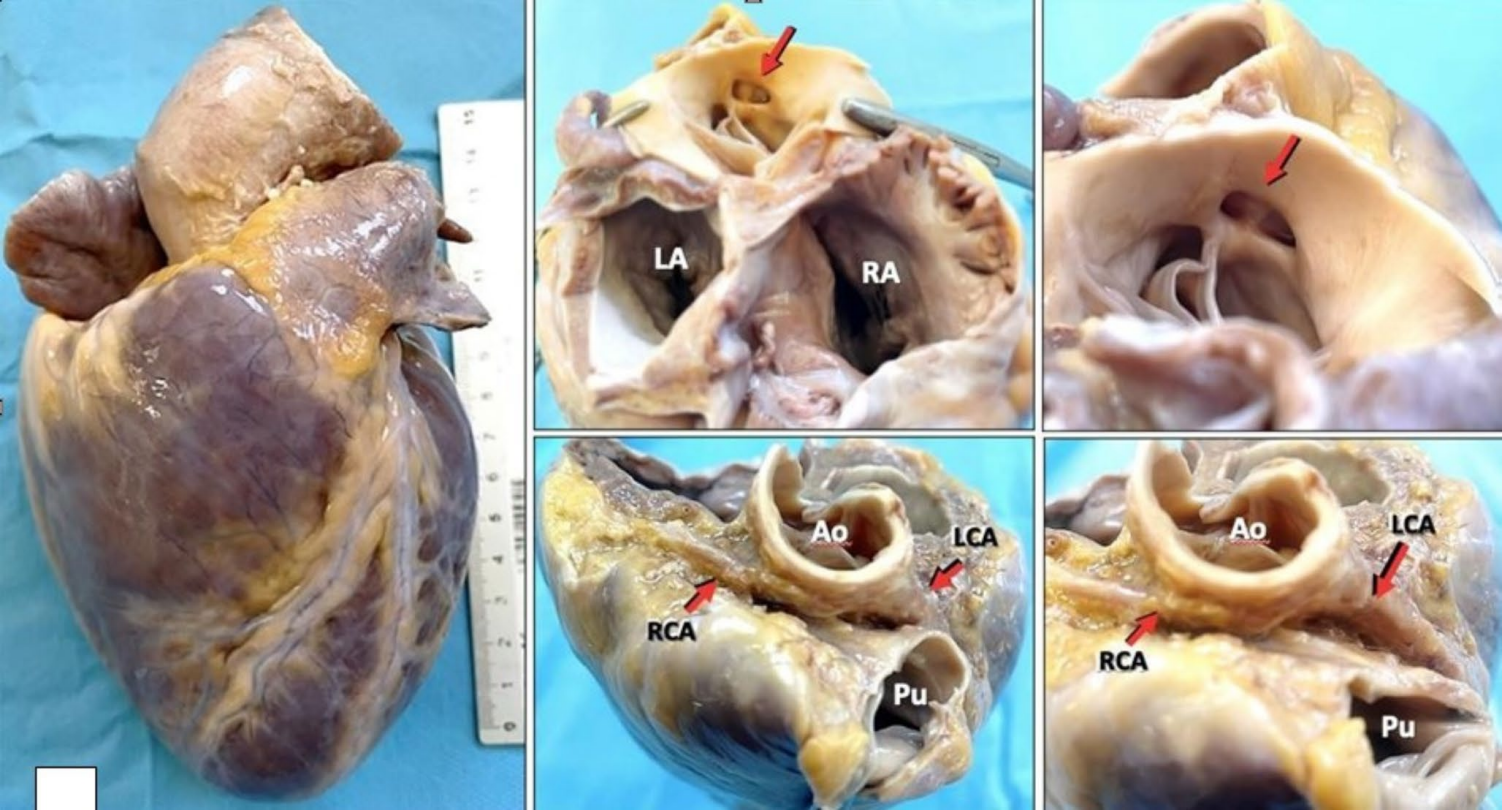
Both coronary arteries originated from a single ostium above the aortic cusps, resulting in an acute angle and slit lumen between the aorta and pulmonary artery (LA: Left Atrium; RA: Right Atrium; LCA: Left Coronary Artery; RCA: Right Coronary Artery; Ao: Aorta; Pu: PulmonaryArtery)

The right ventricle thickness was 8 mm, the left ventricle thickness was 15 mm, and the septum thickness was 18 mm. The foramen ovale was closed. No anomalies were detected in the atrioventricular or semilunar valves. There was no mechanic impedance to left ventricular outflow tract, the papillary muscles insertion to the mitral valve was normal. A single coronary ostium located over the commissure between the left and right aortic cusps was observed. The first section of the left coronary artery and the right coronary artery showed a slit lumen and acute angle from right to left (left coronary artery) and from left to right (right coronary artery), respectively,. The right coronary artery arised form the left sinus of Valsalva, with an interarterial course between the aorta and pulmonary artery, along the atrioventricular space and subsequently in the posterior face of the heart giving the posterior interventricular artery branch (Fig. 1). A dominant right coronary artery was present. A standard sampling of the heart was performed during autopsy (three samples of left ventricle, one septum, one right ventricle, coronary arteries).

**Fig. 2:**
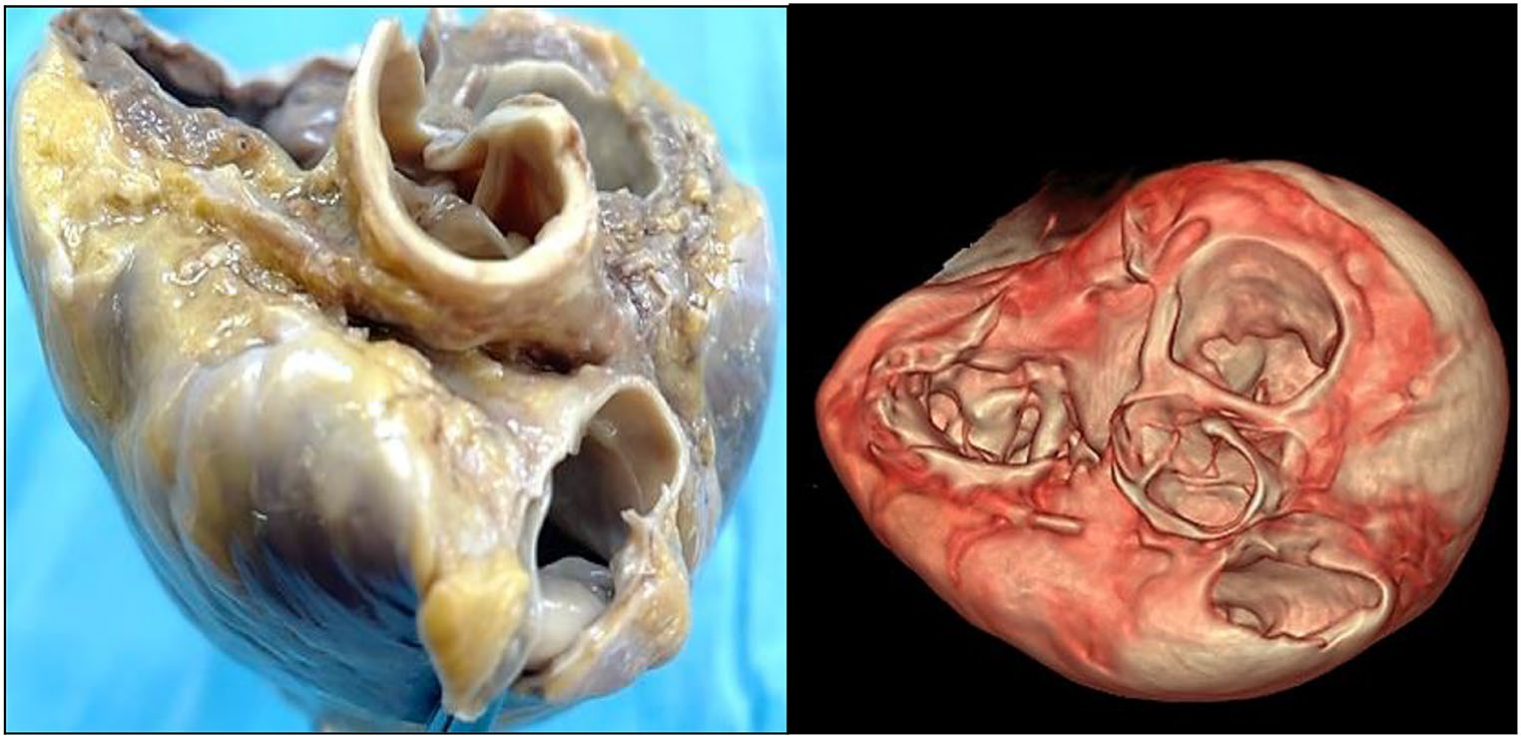
Postmortem magnetic resonance imaging (MRI) confirmed the anomalous origin and course of both coronary arteries. The right coronary artery arised form the left sinus of Valsalva, with an interarterial course between the aorta and pulmonary artery, along the atrioventricular space and subsequently in the posterior face of the heart giving the posterior interventricular artery branch. A dominant right coronary artery was present

Postmortem magnetic resonance imaging (MRI) confirmed the anomalous origin and course of both coronary arteries and the cardiac hypertrophy, with no signs of significant fibrosis in the left and right ventricular walls (Fig. 2).

**Fig. 3:**
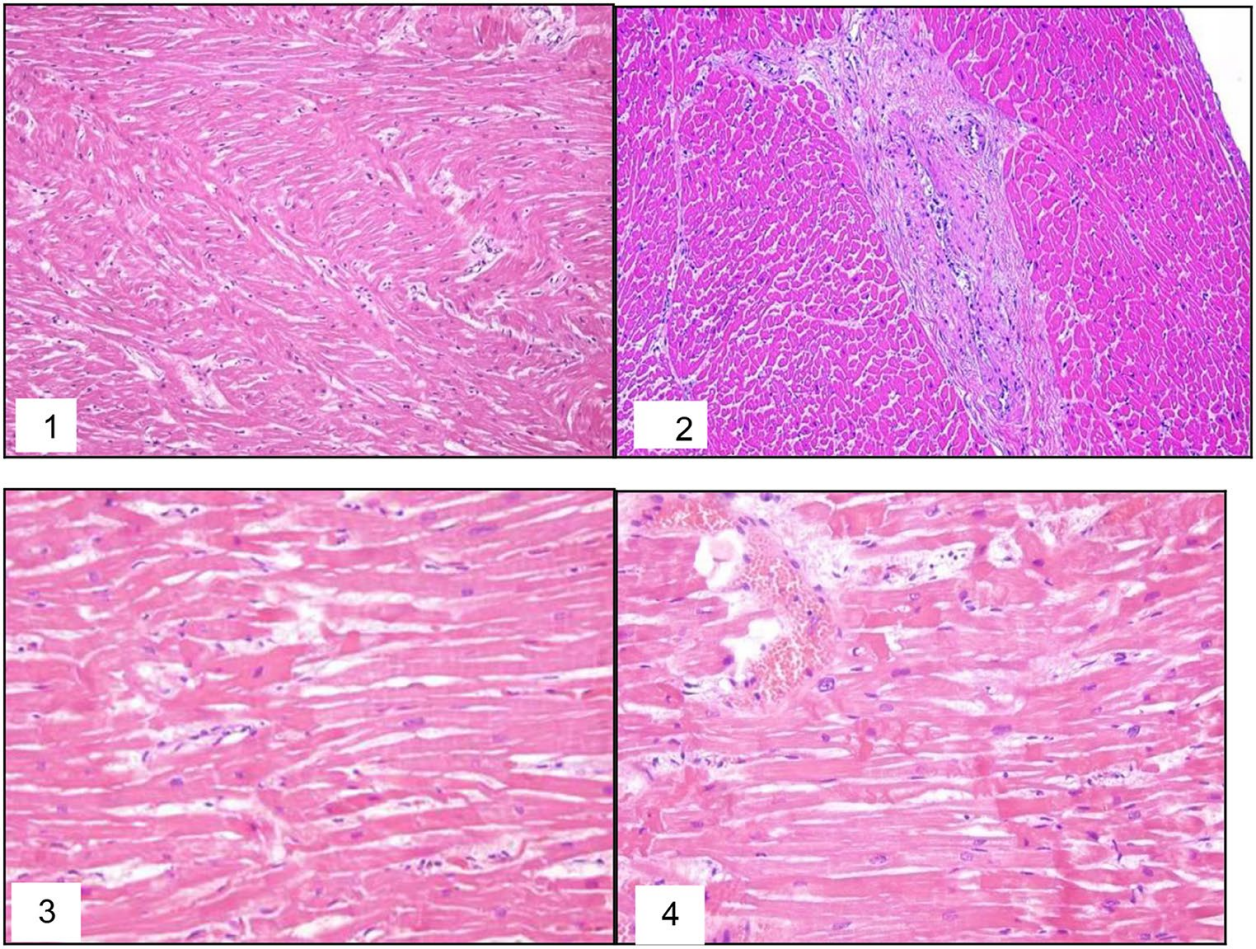
Myocardial disarray in the lateral wall of the left ventricle (1) with fibromuscular dysplasia of small coronary vessels (2) and areas with contraction band necrosis in the posterior wall of the left ventricle and the septum (3–4)

Histopathological examination revealed mild myocardial disarray in left ventricle sample, more prominent in the lateral wall sample, with fibromuscular dysplasia of small coronary vessels and areas with contraction band necrosis in the posterior wall and septum samples (Fig. 3). Light interstitial fibrosis was observed in the posterior left ventricle papillary muscle. Microscopic examination of the other organs was unremarkable.

Toxicological examination of blood and urine samples was negative for common drugs of abuse.

The death was attributed to sudden cardiac death due to hyperkinetic ventricular arrhythmia secondary to myocardial ischemia of the right ventricle wall and septum in a subject with an anomalous origin of both coronary arteries and underlying hypertrophic cardiomyopathy.

## Discussion

Sudden cardiac death (SCD) is one of the most common causes of death, especially in young people [1].

The exact incidence of SCD is unknown because not all individuals with SCD undergo autopsy [2].

As highlighted recently by Banner J et al. [3], in approximately 40% of SCDs occurring in individuals under 50 years of age, autopsy is not performed. Tseng et al. [4] reported that the autopsy rates for out-of-hospital natural deaths range from 10% in the United States to 23% in some European countries. Furthermore, the standard protocol for autopsy examination of SCD developed by the Association for the European Society of Cardiology (AECVP) should be adopted for an accurate diagnosis of SCD and the determination of its causes [3–5].

The AECVP reported an incidence of SCD of between 36.8 and 39.7 for 100,000 individuals per year [3, 6, 7]. In other studies, the reported incidence ranges from 50 to 100 per 100,000 individuals, and the incidence of SCD related to sports in North America and Europe is approximately 15,000 per year [8].

Underlying inherited/congenital cardiac disorders, such as cardiomyopathies, congenital coronary anomalies and ion channelopathies, as the cause of SCD are more common in young athletes than nonathletes [9–12].

Epidemiological studies conducted in northeastern Italy revealed that the incidence of sudden death in athletes is higher than that in nonathletes (2.3/100,000/year versus 0.9/100,000/year) [13, 14].

As highlighted in a previous meta-analysis of postmortem studies, the most common causes of SCD in young athletes are a an abnormal heart structure (19.7%), HCM (14.1%), idiopathic left ventricular hypertrophy (10.6%), and an anomalous origin of the coronary arteries (9.7%) [15].

Among the congenital coronary anomalies, an abnormal right coronary artery origin is more common than an abnormal left coronary artery origin [16]; the ectopic coronary artery originating from the pulmonary artery and an anomalous origin of a coronary artery from the wrong sinus of Valsalva, particularly when the course is intramural in the first tract, have been considered risk factors for sudden cardiac death [2, 17–19].Congenital anomalies of coronary arteries are uncommon diseases that occur in 0.2–1.2.% of the population [20]. Although uncommon diseases, congenital coronary anomalies account for 15–25% of SCD cases among athletes [21]. Right coronary artery originating from the left sinus of Valsalva is associated with SCD. Taylor et al. [22] demonstrated that 13 of 52 patients with anomalous origin of right coronary artery experienced sudden cardiac death with no previous symptoms. The compression of the segment of an anomalous right coronary artery originating from the left sinus of Valsalva between the aorta and the pulmonary artery is a significant mechanism of coronary compromise [21]. This anatomical anomaly can lead to dynamic systolic compression, luminal narrowing, and impaired coronary perfusion, especially during exertion, increasing the risk of myocardial ischemia and sudden cardiac death (SCD).

In most patients, origin anomalies are silent in clinical testing (standard 12-lead ECGs), with results remaining within normal limits, and these anomalies are often first detected at autopsy [1, 23, 24].Among different sports, basketball and football are particularly high-risk sports [1].

The grass anatomy and histopathological finding of the heart revealed a hypertrophic cardiomyopathy (HCM). In the 2007 the European Society of Cardiology working group on myocardial and pericardial diseases with a position statement on the classification of the cardiomyopathies established that “hypertrophic cardiomyopathies are simply defined by the presence of increased ventricular wall thickness or mass in the absence of loading conditions (hypertension, valve disease) sufficient to cause the observed abnormality” [25]. This approach avoids to relate HCM to one phenotype and etiology only (genetic determined sarcomeric protein disease) [25]. The Clinical diagnosis of HCM is based on the presence of hypertrophy – usually identified with cardiac imaging tests – not related to another cardiac, systemic, metabolic, or syndromic disease. Left ventricular thickness is 15 mm or more, with several phenotype characterized by different asymmetric patterns of hypertrophy [26]. A typical feature is the obstruction in the left ventricular outflow tract, described in 70% of the cases and frequently related to arrhythmic events [26]. A recent autopsy study based on 86 SCD cases due to HCM diagnosed at autopsy only on increased left ventricular (LV) wall thickness (≥ 13 mm), with nondilated LV in the absence of other cardiac or systemic disease, examined the relationship between HCM and sudden cardiac death [27]. The HCM histopathological findings in this autopsy series were: myocyte disarray (88%), thickening of intramural arterioles (56%), myocardial fibrosis (70%), replacement scars (13%) coronary artery bridging (n 3), coronary art ery narrowing (> 75%) [27]. In the presented case the diagnosis of HCM was performed based on the symmetric increased LV and septum thickness (15 mm and 18 mm respectively), the presence of foci of disarray, the increased heart weight according to age, a negative history of high-intensity and habitual physical activity, and the exclusion of other cardiac or non-cardiac causes of cardiac hypertrophy. No genetic tests were performed. However, the relationship between HCM phenotype and SCD was excluded. No signs of fibrosis were present except for light physiological fibrosis in the posterior left ventricle papillary muscle. There were no signs of left ventricle outflow tract obstruction (mitral valve damage and septal fibrosis). Myocardial disarray was not diffuse involving mainly the lateral wall of the left ventricle. Meanwhile, in the myocardial territory supplied by the right coronary artery (1/3 posterior of interventricular septum and posterior left ventricle wall) focal areas with contraction band necrosis (typical acute signs of ischemia) were detected. No other ischemic areas were observed in the myocardium. The course of the coronary artery along the interarterial space, the histopathological findings and the concomitant physical activity led to assess the mechanism of death.

In the reported case, the cause of death was attributed to hyperkinetic ventricular arrhythmia during sport activity triggered by myocardial ischemia due to obstruction of the interarterial tract of right coronary artery caused by its anomalous origin and course. Autopsy studies on coronary artery anomalies play a crucial role in advancing our understanding of the pathological anatomy of these conditions, their association with sudden cardiac death (SCD), and the underlying mechanisms leading to fatal events. As highlighted in Angelini at al. study [28], novel imaging techniques have improved the ability to assess the prevalence of coronary anomalies, their clinical significance, and their potential to cause life-threatening complications. However, postmortem investigations remain essential to validate imaging findings, clarify mechanisms of death, and refine risk stratification. By correlating anatomical anomalies with SCD, autopsy studies provide critical insights that support the development of effective screening strategies, facilitate early diagnosis, and promote preventive measures to reduce the incidence of sudden cardiac events in at-risk individuals. 

## Key points

1. Autopsy is mandatory in case of sudden death in the young to underline the mechanism o death and implement the knowledge on congenital coronary anomalies.
2. Cardiac Pathology expert evaluation is needed in case of Sudden Cardiac Death in the young 3.Postmortem MRI is an helpful tool for the diagnosis of coronary arteries anomalies.

## Data Availability

The data presented in this study are available on request from the corresponding author.
